# Dual-Responsive Core Crosslinking Glycopolymer-Drug Conjugates Nanoparticles for Precise Hepatocarcinoma Therapy

**DOI:** 10.3389/fphar.2018.00663

**Published:** 2018-07-17

**Authors:** Jing Wu, Jiayi Yuan, Baotong Ye, Yaling Wu, Zheng Xu, Jinghua Chen, Jingxiao Chen

**Affiliations:** Key Laboratory of Carbohydrate Chemistry and Biotechnology, Ministry of Education, School of Pharmaceutical Sciences, Jiangnan University, Wuxi, China

**Keywords:** glycopolymer, redox-responsive, pH-sensitive, hepatocarcinoma therapy, ASGPR

## Abstract

Nanoparticles (NPs) have demonstrated a potential for hepatocarcinoma therapy. However, the effective and safe NP-mediated drug transportation is still challenging due to premature leakage and inaccurate release of the drug. Herein, we designed a series of core cross-linking galactose-based glycopolymer-drug conjugates (GPDs) NPs with both redox-responsive and pH-sensitive characteristics to target and program drug release. Glycopolymer is comprised of galactose-containing units, which gather on the surface of GPD NPs and exhibit specific recognition to hepatocarcinoma cells, which over-express the asialoglycoprotein receptor. GPD NPs are stable in a normal physiological environment and can rapidly release the drug in hepatocarcinoma cells, which are reductive and acidic, by combining disulfide bond cross-linked core, as well as boronate ester-linked hydrophilic glycopolymer chain and the hydrophobic drug.

## Introduction

Human hepatocellular carcinoma (HCC) is a common type of primary liver cancer and is diagnosed in more than half a million people worldwide (de Souza et al., 2015). At present, HCC has received increasing attention because of its important effect on the physiological functions of the liver, high lethality, and the growing incidences in many regions (Torre et al., 2015). Chemotherapy is a common HCC treatment strategy that is limited because it is always accompanied by dose-limiting toxicity, high rate of tumor recurrence, and drug resistance (Dutta and Mahato, 2017; Kuruvilla et al., 2017). Moreover, the phagocytosis of Kupffer cells hinders the accuracy and efficacy of chemotherapeutics (Lahmar et al., 2016). To date, nanotechnology is applied to address these problems and has obtained effective molecular-level diagnosis (Mura et al., 2013; Lim et al., 2015). It also serves as vectors, sensors, and targeting agents to achieve optimal efficacy in precise drug transportation because of its potential to alter the biodistribution and pharmacokinetics of drugs (Tao et al., 2013, 2015, 2016; Li et al., 2016; Zhang et al., 2017; Dai et al., 2018). In addition, some nanomaterials, which are known as theranostic nanosystem, provide a novel approach to obtain ideal efficacy by combining diagnosis and treatment together (Tao et al., 2017a,b; Zhu et al., 2018). However, the clinical application of the nanoparticle (NP)-mediated treatment is still challenging due to the premature drug leakage and inaccurate drug release in dilute bloodstream, thereby resulting in serious systemic side-effects to normal tissues and cells (Tao et al., 2014; Tibbitt et al., 2016; Chen et al., 2017; Rosenblum et al., 2018).

Over the past decades, neoplasm pathophysiology gradually reveals distinctive hallmarks of the tumor from normal tissues (Hanahan and Weinberg, 2000; Hanahan and Weinberg, 2011; Flavahan et al., 2017). To the best of our knowledge, hepatic galactose/*N*-acetylglucosamine receptor, also known as asialoglycoprotein receptor (ASGPR), is specifically exposed on the surface of hepatoma cells with a considerably high amount (Rigopoulou et al., 2012; Lepenies et al., 2013). Thus, ASGPR is used as an autoantigen to achieve targeted hepatopathy therapy through specific recognition of galactose (Fu et al., 2015). Moreover, tumor microenvironment, such as acidity (Dong et al., 2013; Du et al., 2015), hypoxia (Wilson and Hay, 2011; Semenza, 2017), high level of glutathione (GSH) (Dutta et al., 2017), and overexpressed enzymes (Zhu et al., 2012; Chen et al., 2015), inspires rational design of smart NPs to respond to various biochemical and physicochemical stimuli (Fleige et al., 2012). To achieve this goal, researchers introduced cleavable linkages (Ma and Tian, 2014), such as pH-sensitive bonds [e.g., boronate ester (Liu et al., 2013; Chen et al., 2016) and “Schiff” base (Chen et al., 2014)] and redox-responsive linkages [e.g., disulfide (Yu et al., 2014; Xu et al., 2017) and Se–Se bonds (Xu et al., 2013)] to construct NPs, which not only distinguishes carcinoma cells from normal cells but also regulates the drug release procedure and precisely meets the mechanisms of various agents, such as extracellular and intracellular release (Ding et al., 2017; Shi et al., 2017). Therefore, to realize a reliable and efficient NP-mediated HCC treatment, stable loading of hydrophobic agents in the blood circulation without leakage, selective transport, and release of the drug into hepatoma cells are necessary.

**FIGURE 1:**
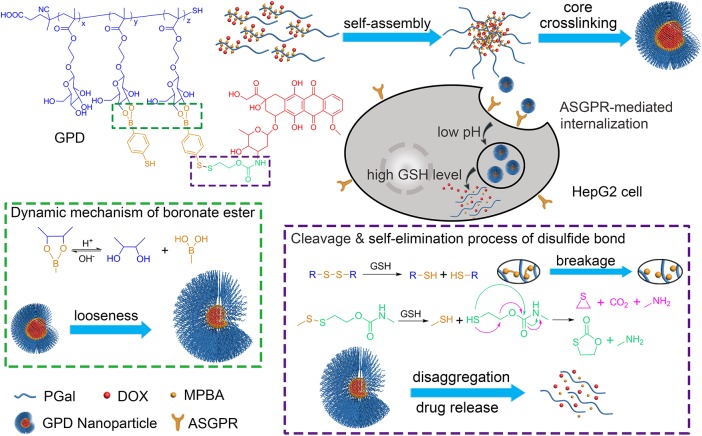
Construction of galactose-based glycopolymer-DOX conjugates (GPDs) NPs for selective HCC therapy. The redox- and pH responsive mechanisms were designed to achieve program drug release in cancer cells.

Herein, we developed a core cross-linking glycopolymer-drug conjugates (GPDs) NPs with unique dual-responsive characteristics to achieve selective transportation and program release of anticancer drug for HCC treatment (**Figure 1**). Inspired by the specific recognition between ASGPR and galactose (D’Souza and Devarajan, 2015), and the cluster glycoside effect (Dimick et al., 1999; Lundquist and Toone, 2002), which can effectively improve the affinity of carbohydrate ligands for their protein receptors, we employed galactose to build glycopolymer to obtain enhanced ASGPR-mediated hepatoma cellular binding and internalization. A disulfide bond was introduced to the side-chain of glycopolymer via a dynamically covalent boronate ester between galactose moieties and phenylboronic acid, which exhibits pH-regulated characteristics. Subsequently, hydrophobic model anticancer drug doxorubicin (DOX) was conjugated with the glycopolymer to form a series of amphiphilic conjugates through a self-eliminating disulfide bond (Satyam, 2008; Roy et al., 2015). This process ensures the traceless release of DOX, thereby keeping the original chemical structure and pharmacological action of DOX. Moreover, the hydrophobic core of the self-assembly GPD NPs was interiorly cross-linked through disulfide bond to stabilize the architecture and avoid drug leakage in the physiological environment. The GSH level in the cytosol of cancer cells is much higher than that in normal cells or extracellular fluid (Wang et al., 2013). Thus, by incorporating both redox-responsive and pH-sensitive characteristics into the GPD NPs, DOX can be accurately and programmatically released from the NPs in the cytoplasm of hepatoma cells, which are reductive and acidic.

## Materials and Methods

### Materials

β-*D*-Galactose pentaacetate (98%) was purchased from Alfa Aesar (China) Chemical Co. Ltd. 2-Hydroxyethyl methacrylate (HEMA, 97%) was obtained from J&K Scientific Ltd. (China). Boron trifluoride diethyletherate (BF_3_⋅Et_2_O, 98%) was purchased from Aladdin Reagent (Shanghai) Co., Ltd. 4-Cyano-4-(phenylcarbonothioylthio)pentanoic acid (CPA, >97%), GSH, and 2,2′-Azobis(2-methylpropionitrile) (AIBN) were purchased from Sigma-Aldrich (China) Inc. 4-Mercaptophenylboronic acid (MPBA, >95%) was obtained from Energy Chemical Co. (China). DOX hydrochloride (DOX⋅HCl, 99%) was provided by Beijing HvsF United Chemical Materials Co., Ltd. (China) and used as received. AIBN was recrystallized from ethanol before use. All other chemical regents and solvents were purchased from Shanghai Chemical Reagent Co. (China) and of analytical reagent grade, used directly. Dulbecco’s modified Eagle’s medium (DMEM), Roswell Park Memorial Institute (RPMI) 1640 medium, fetal bovine serum (FBS), penicillin-streptomycin, and Lysotracker green DND-26 were purchased from Thermo Fisher Scientific (United States). 3-(4,5-dimethylthiazol-2-yl)-2,5-diphenyltetrazolium bromide (MTT), trypsin, and 4,6-diamidino-2-phenylindole (DAPI) staining solution were obtained from Beyotime Biotechnology Co., Ltd. (China).

### Synthesis of 2-[(2,3,4,6-tetra-*O*-acetyl-β-*D*-galactopyranosyl)oxy]ethyl Methacrylate (GalAc-EMA)

β-*D*-galactose pentaacetate (5.0 g, 13 mmol) and HEMA (1.3 ml, 10 mmol) were dissolved in 50 ml dichloromethane (DCM) in an ice bath under N_2_ atmosphere, and BF_3_⋅Et_2_O (4.4 ml, 34 mmol) was added dropwise into the solution. After stirring at 0°C for 2 h, the mixture was moved to room temperature continued to be stirred overnight. Afterwards, the suspension was filtrated and the filtrate was washed with DI water and saturated NaCl solution for three times, then, dried over anhydrous magnesium sulfate. The product was obtained and purified by column chromatography (silica gel, DCM/ethyl acetate, 4/1). Yield: 73% (light yellow viscous liquid). The chemical structure of GalAc-EMA was identified with ^1^H NMR spectrum on an AVANCE III (Bruker, Germany) equipment (Supplementary Figures 1, 2).

### Synthesis of polyGal-EMA (PGal)

GalAc-EMA (1.00 g, 2.17 mmol), CPA (24.3 mg, 0.09 mmol), and AIBN (2.8 mg, 0.02 mmol) were dissolved in 2 ml anhydrous dimethyl sulfoxide (DMSO). The mixture was degassed with a freeze-vacuum-thaw cycle for three times under N_2_ atmosphere. Then, the reaction was stirred at 75°C for 24 h. Afterwards, the mixture was cooled down and the product (PGalAc) was precipitated in cold diethyl ether. The product was collected by centrifugation and dried under vacuum. Yield: 92% (pink solid, Supplementary Figure 3).

PolyGal (PGal) was obtained by deprotecting the acetyl group of PGalAc. Briefly, 0.5 g of PGalAc was dissolved in 50 ml DCM, and 1 ml CH_3_OH solution of CH_3_ONa (30%) was added dropwise. After stirring at room temperature for 20 min, the solution was adjusted to neutral pH using dilute HCl (1 M). The solution was then dialyzed with DI water for 48 h, and the PGal was collected by freeze-drying. Yield: 63% (white solid, Supplementary Figure 4).

### Synthesis of Glycopolymer-DOX Conjugates (GPDs)

PolyGal (45 mg, 0.1 mmol) was dissolved in 5 ml anhydrous DMSO and the solution was degassed with N_2_ for 30 min. Then, 5 ml degassed DMSO solution of MPBA and disulfide-activated DOX (DOX-ss-Py), the synthesis route and ^1^H NMR spectrum (shown in Supplementary Figures 5, 6) was added dropwise. The molar ratio of PGal, MPBA, and DOX-ss-Py was adjusted to obtain a series of GPDs with different hydrophilic/hydrophobic balances (**Table 1**). The mixture was stirred at room temperature for 12 h and poured into an ammonium bicarbonate solution (pH 8.5). After dialyzing in the dialysis tube (molecular weight cut-off, MWCO: 3.5 kDa) with ammonium bicarbonate solution for 24 h, the product was collected by freeze-drying, and the degrees of substitution (DS) of the pendant group were evaluated from ^1^H NMR spectra (Supplementary Figures 7, 8). In addition, DOX was directly connected with PGal through succinic anhydride to form a covalent linked PGal-DOX conjugate (GDC) as the control. The drug loading (DL) amount of GPDs was determined using UV–vis spectrophotometer on a UV-2550 (Shimadzu, Japan) equipment at 480 nm in DMF, and the DL values were calculated as follows: DL (%) = (mass of DOX) × 100/(mass of GPDs).

**Table 1:** Characterizations of GPDs and GPD NPs.

GPD	Molar ratio of units Gal-EMA/MPBA/DOX		GPC measurements			DL (wt%)	TEM estimated size (nm)	DLS measurements		
					
	Feeding ratio	Calculated ratio^a^	*M*_w_ (Da)	*M*_n_ (Da)	PDI			Size (nm)	PDI	ζ-potential (mV)
GPD1	10:3:1	10.4:3.2:1	10,115	7964	1.27	8.9	176.3 ± 10.5	279.7	0.41	0.01
GPD2	7:2:1	7.4:2.3:1	10,879	9142	1.19	10.2	107.3 ± 6.6	134.1	0.21	-0.03
GPD3	7:3:1	7.2:3.3:1	11,559	9796	1.18	12.4	98.6 ± 7.9	113.8	0.14	0.02
GPD4	8:3:2	4.2:1.2:1	12,643	10,279	1.23	16.5	21.4 ± 3.9	59.2	0.16	0.00


**^*a*^The ratio was calculated form the characteristic peak area integration of ^*1*^H NMR spectra.**

### Preparation and Characterizations of GPD NPs

Glycopolymer-drug conjugates were dissolved in phosphate buffer solution (PBS, pH 7.4) at different concentrations and incubated at 37°C for 1 h to allow the self-assembly. The concentration of GPDs was optimized to be 0.5 mg/ml under the evaluation of average size and size distribution of the formed GPD NPs by dynamic light scattering (DLS) technique using a Zetasizer Nano ZS (Malvern, United Kingdom) apparatus at 37°C. The stability of GPD NPs was estimated by recording the variation of size distribution at different times in the PBS (pH 7.4 or 5.5) with or without GSH (10 mM) at 37°C. The morphology of the GPD NPs was observed by transmission electron microscopy (TEM) on a JEM-2100 (JEOL, Japan) instrument with an acceleration voltage of 200 kV. The samples were prepared by dripping a drop of solution onto a copper grid, dried naturally and then followed by negatively staining with phosphotungstic acid solution (0.2%, w/v), and dried in the air.

### *In Vitro* Drug Release Assay

The solution of GPD3 NPs (1 mg/ml, 2 ml) was put into dialysis tubes (MWCO: 3.5 kDa) and immersed into 10 ml of PBS (pH 7.4 or 5.5, with or without 10 mM of GSH) at 37°C, respectively, to simulate the drug release behavior in different physiological conditions. After each sampling at the assigned time intervals, the buffer was replaced with the corresponding fresh medium. The amount of released drug in the medium was determined by UV–vis spectrophotometer at 480 nm. The cumulative release ratio of drug (%) = (mass of released drug) × 100/(mass of total drug). Each value was averaged from three independent trials. The GDC NPs and DOX non-covalently loaded GDC (DOX@GDC) NPs were used as the control.

### Cell Culture

Human hepatocyte carcinoma cell line (HepG2 cells) and transformed African green monkey SV40-transformed kidney fibroblast cell line (COS7 cells) were incubated in DMEM complete medium, and human gastric adenocarcinoma cell line (MGC-803 cells) was incubated in RPMI 1640 complete medium at 37°C in a humidified atmosphere containing 5% CO_2_. The medium contains 10% FBS and 1% (penicillin–streptomycin, 100 U/ml).

### Cytotoxicity Assay *in Vitro*

The cytotoxicity of GPD3 NPs was performed against HepG2, MGC-803, and COS7 cells by MTT assay. Briefly, cells were seeded in 96-well plates at a density of 5000 cells per well with 100 μl of complete culture medium. After cells were cultured to the logarithmic phase, the solutions of GPD3 NPs with various concentrations were added into the well. The cells were cultivated for 48 h, and then, the culture medium was replaced with 100 μl of MTT solution (0.5 mg/ml in PBS) and incubated at 37°C for 4 h. The medium was removed and 150 μl of DMSO was added to each well for dissolving the formazan. The optical density (OD) was measured at 570 nm using Multiskan MK3 microplate reader (Thermo, United States). The relative cell viability was calculated as follows: Cell viability (%) = OD_sample_ × 100/OD_control_, each date was obtained from the average value of three independent trials. The cytotoxicity induced by DOX⋅HCl was measured as the positive control, using 5% DMSO as the co-solvent.

### Confocal Laser Scanning Microscope (CLSM) Observation and Flow Cytometry Analysis

HepG2, MGC-803, and COS7 cells were seeded in confocal dishes at 1 × 10^5^ cells per well, respectively. Then, each type of cells was incubated with GPD3 NPs (equivalent to 10 mg/l free DOX) for 2 and 4 h. The cells were carefully washed with PBS three times and fixed with 4% paraformaldehyde for 15 min. Subsequently, the cells were stained with DAPI (0.2 μg/ml) for 30 min and washed with PBS three times. Afterwards, the cells were viewed under a TCS SP8 confocal laser scanning microscope (CLSM, Leica, Germany).

For the quantitative analysis, three types of cells were seeded in six-well plates and incubated for 24 h, and the medium was replaced with the medium solution of GPD3 NPs (equivalent to 5 mg/l free DOX). After incubating for 1, 2, and 4 h, the cells were washed with PBS (pH 7.4) carefully for three times and collected by trypsinization. Then, the cells were resuspended in 0.5 ml of PBS (pH 7.4) and quantitatively analyzed by flow cytometry, using FACSCalibur flow cytometer (BD Biosciences, United States). The number of cells collected was 20,000, and each experiment was performed with three independent trials. To identify the ASGPR-mediated internalization of GPD3 NPs, HepG2 cells were pre-incubated with 0.5 ml of galactose solution (2 mg/ml) or PGal (the monomer units amount was equivalent to 2 mg/ml of galactose). After 2 h incubation, the GPD3 NPs solution (equivalent to 5 mg/l free DOX) was directly added into the plate. The cells were continuously incubated for 2 or 4 h. Then, the plate was carefully washed with PBS (pH 7.4) and the mean fluorescent intensity (MFI) was measured by flow cytometer.

### Intracellular Distribution Assay

HepG2 cells (4 × 10^4^ cell/well) were seeded in dishes and incubated in RPMI 1640 (1 ml) containing 10% FBS for 24 h. Then, GPD3 NPs (equivalent to 5 mg/l DOX) dispersed in the culture medium were added and the cells were incubated at 37°C for 2, 4, and 8 h. After removing the medium and washing with PBS (pH 7.4) three times, the cells were fixed with 4% paraformaldehyde. Afterwards, the cells were successively stained with 0.5 ml of LysoTracker-Green DND-26 (50 nM) for 30 min and 0.5 ml of DAPI Solution (0.2 μg/ml) for 15 min. Then, the cells were carefully washed with PBS and observed by CLSM. Free DOX was employed as the positive control and incubated for 4 h.

## Results and Discussion

### Preparation and Characterizations of GPDs

First, GalAc-EMA and DOX-ss-Py were successfully synthesized (Kularatne et al., 2010; Kumar et al., 2015), and their chemical structures were identified with ^1^H NMR spectra (see details in the Supporting Information). Then, we employed a classical reversible addition-fragmentation chain transfer (RAFT) polymerization method and used GalAc-EMA as the monomer to synthesize a galactose-based glycopolymer, PGal. Subsequently, thiol group was introduced into the side-chain of PGal through a dynamically covalent interaction between a diol group of galactose units and a phenylboronic acid group of MPBA in a mildly alkaline condition (Deshayes et al., 2013). Afterward, DOX was connected with PGal through a designed disulfide bond, which is a redox-responsive linkage with self-eliminating characteristic (Santra et al., 2011; Li et al., 2015). A series of GPDs were received by adjusting the molar ratio of three units, that is, Gal-EMA, MPBA, and DOX. Moreover, the amount of MPBA was higher than that of DOX moiety in our design to set aside a portion of thiol group for disulfide cross-linking. As shown in **Table 1**, the molar ratios of three units of GPDs calculated from their characteristic peak area integrations of ^1^H NMR spectra (Supplementary Figure 8) exhibited close values in comparison with their feeding ratios. These results indicated that the components of GPDs were easy to be tuned by varying the feeding ratio of the three units. In addition, GPC measurements indicated that the molecular weight of four GPDs were close to 10 kDa with narrow distributions, where their polydispersity values were lower than 1.3. These results showed a controllable nature of RAFT polymerization. The DL values of four GPDs measured using the UV–vis spectroscopy were 8.9, 10.2, 12.4, and 16.5%. These values were consistent with the results calculated from the ^1^H NMR. Among the four GPD samples, GPD3 and GPD4 possessed higher content of DOX than the other two samples. Thus, their molecular weights were relatively higher than that of GPD1 and GPD2. Theoretically, these GPDs exhibited amphiphilic characteristic because galactose is hydrophilic, while DOX moieties show hydrophobic nature. These results demonstrated the hydrophilic/hydrophobic balance of the designed GPDs can be adjusted by varying the molar ratio of three units, which regulated the self-assembly behavior of GPDs to some extent.

### Morphology of GPD NPs

**FIGURE 2:**
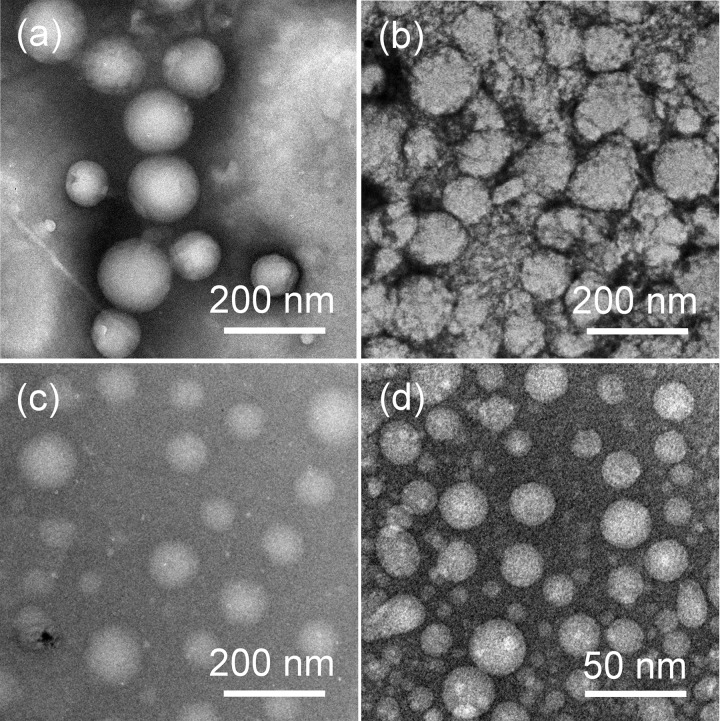
Transmission electron microscopy (TEM) images of self-assembly **(a)** GPD1, **(b)** GPD2, **(c)** GPD3, and **(d)** GPD4 NPs in PBS (pH 7.4).

After synthesis, we separately dissolved four GPDs in PBS (pH 7.4) to allow the self-assembly of GPDs to form various NPs. As shown in **Figure 2**, all four GPDs formed homogeneous spherical NPs in an aqueous solution. The mean diameter of four GPDs estimated from the TEM images were 176.3 ± 10.5, 107.3 ± 6.6, 98.6 ± 7.9, and 21.4 ± 3.9 nm (**Table 1**). As discussed above, the self-assembly of GPDs was regulated by varying their hydrophilic/hydrophobic balance depending on the proportions of MPBA and DOX moieties in the GPDs. The average size of GPD NPs showed decreasing trend with increasing amount of MPBA and DOX. Among the four samples, GPD3 and GPD4 have higher DOX contents and stronger hydrophobicity, thereby leading these two GPDs to form more uniform NPs than the other samples. This phenomenon is attributed to the fact that hydrophobic moieties, that is, MPBA and DOX, gathered at the core region and drive self-assembly to form NPs. Thus, the increasing hydrophobicity of GPDs increased the kernel density, reduced the critical aggregation concentration, and stabilized the architecture of NPs, thereby resulting in the decrease in particle size. Additionally, the average sizes of four GPD NPs measured from DLS were 279.7, 134.1, 113.8, and 59.2 nm (Supplementary Figure 9), thereby showing comparable results with those of TEM observation. DLS measured average size was a litter higher than the estimated average size from the TEM images. However, the difference was reduced with the increasing amount of MPBA. These results were attributed to the thiol group on MPBA charge of the core cross-linking. Thus, the increasing amount of MPBA improved the stability of core region of GPD NPs, thereby resulting in the decreasing difference of the size between hydration state for DLS and dry state in TEM observation. In addition, the ζ-potential value of four GPD NPs exhibited that their surface charges were nearly neutral, thereby indicating that hydrophilic galactose moieties coated on the surface of these NPs. Considering the DOX loading amount and the average size in aqueous medium, we chose GPD3 as the sample for the next study.

### Stability and Environmental Sensitivity of GPD NPs

**FIGURE 3:**
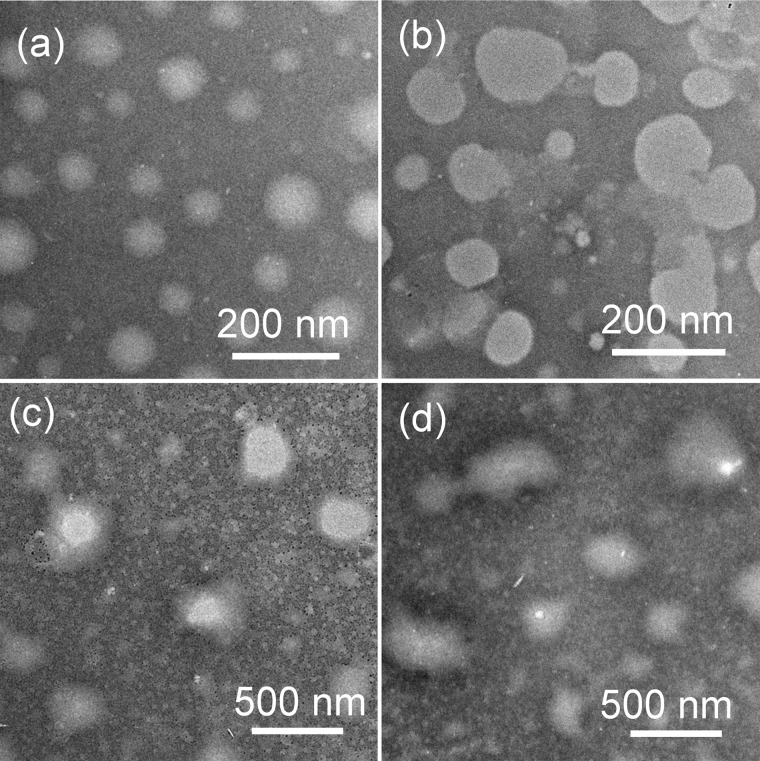
Transmission electron microscopy (TEM) images of the morphologic variation of GPD3 NPs after immersing in **(a)** PBS (pH 7.4), **(b)** PBS (pH 7.4) containing GSH (10 mM), PBS (pH 5.5) **(c)** without, or **(d)** with GSH (10 mM) for 6 h.

**FIGURE 4:**
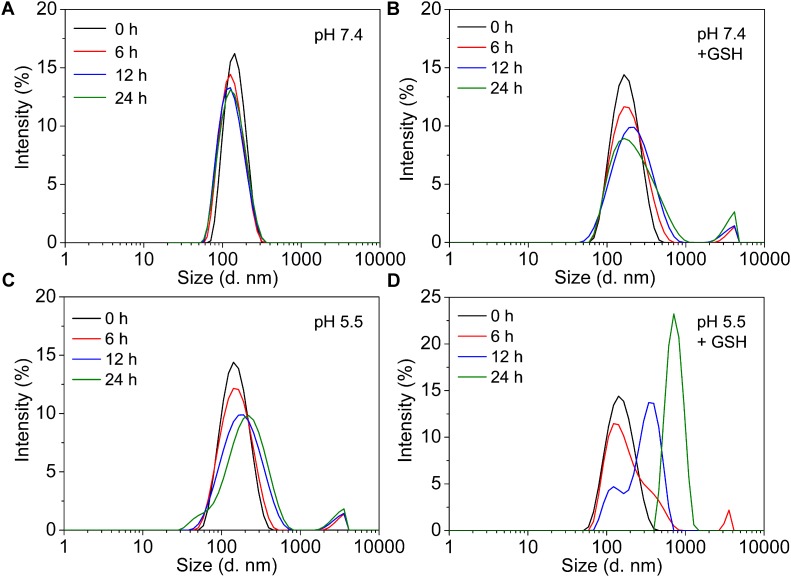
The variation of size distributions of GPD3 NPs after immersing in **(A)** PBS (pH 7.4), **(B)** PBS (pH 7.4) containing GSH (10 mM), PBS (pH 5.5) **(C)** without, or **(D)** with GSH (10 mM) for different periods.

The stability and sensitivity of the self-assembly GPD NPs, which are relative to the long-term circulation and programmed drug transportation, are important for the clinical trial of polymer-drug conjugates. Thus, we evaluated the stability of GPD3 NPs in different milieus to simulate the process in various physiological environments. As shown in **Figure 3**, the TEM images illustrated that GPD3 NPs kept their morphologies in PBS (pH 7.4, **Figure 3a**) but gradually disaggregated in reductive environment (**Figure 3b**), acidic milieu (**Figure 3c**), or the condition with both reductive and acidic characteristics (**Figure 3d**). NPs were increased in size from approximately 100 nm in PBS (pH 7.4) to approximately 200 nm in a reductive solution and continuously increased to 500 nm or more in acidic mediums and acidic buffers containing 10 mM GSH. These results demonstrated that GPD3 NPs exhibited different disaggregation rates and responsiveness levels with a variation of environments. We further employed DLS to detect the disaggregation process of GPD3 NPs in these aforementioned environments, as shown in **Figure 4**. GPD3 NPs exhibited a stable narrow size distribution in PBS (pH 7.4) after 24 h (**Figure 4A**), and this situation lasted for 2 weeks during the measurement (Supplementary Figure 10), thereby showing an outstanding stability of GPD3 NPs. However, the size of GPD3 NPs varied with the alternative environments, showing comparable regular feature with the results illustrated in TEM images. In the reductive and alkalescent solutions, GPD3 NPs loosened, and their average size increased with increasing time (**Figure 4B**) because of the breakage of disulfide bond, which is responsible for core cross-linking of GPD NPs. This process was similar to that in the acidic medium (**Figure 4C**), which leads to the fracture of boronate ester. Moreover, the size was increased intensively in the environment possessing both reducibility and acidity (**Figure 4D**) than in the above-mentioned two conditions. This variation was derived from our designed dual responsive strategy, which obtained precise drug release. In the inner hydrophobic core of GPD NPs, DOX was connected to PGal through disulfide bond and boronate ester. Between the hydrophobic core and hydrophilic shell, excess disulfide bond was employed to cross-link the core region. Thus, GPD NPs showed favorable stability in PBS (pH 7.4), which simulates normal physiological environment. Given that disulfide bond shows responsivity to reductive condition (Yu et al., 2014; Xu et al., 2017), and boronate ester is sensitive to low pH (Chen et al., 2016), GPD NPs became far more unstable in the environment with both reductive and acidic conditions, corresponding to the cytoplasmic and endosomal environment of cancer cells. Therefore, the designed GPD NPs may exhibit a rapid disaggregation in the simulated environment in the cancer cells, which are reductive and acidic, thereby showing programmed drug release feature.

### *In Vitro* Drug Release Assay

**FIGURE 5:**
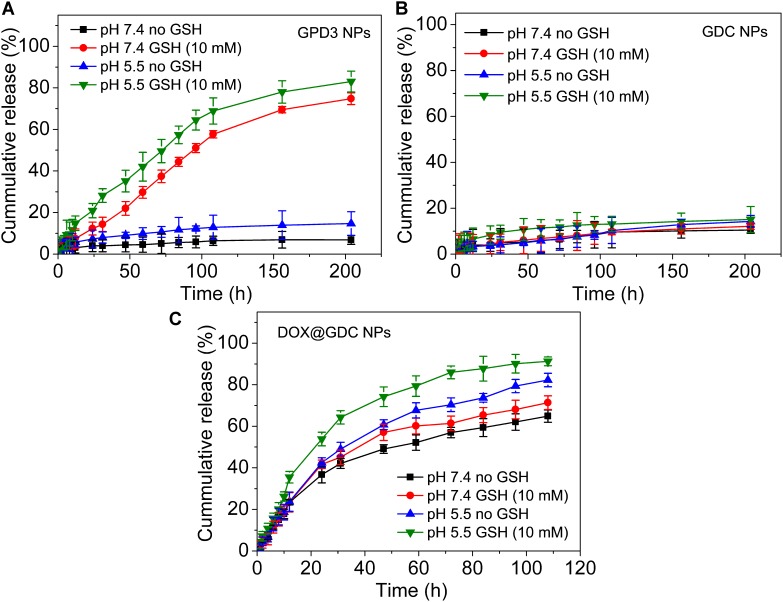
*In vitro* DOX release profiles of **(A)** GPD3 NPs, **(B)** GDC NPs, and **(C)** DOX@GDC NPs in different conditions simulated various physiological environments.

To evaluate the stimuli-responsive drug release of GPD3 NPs, we investigated the DOX release in four different conditions to simulate physiologically biochemical milieus. As shown in **Figure 5A**, GPD3 NPs exhibited different release characteristics in four different conditions. In normal physiological and low-pH conditions, the DOX release rate was lower than 20% for over 200 h. However, the DOX release rate was rapidly increased to more than 70% in the medium containing GSH to induce reductive environment. In particular, the release rate exhibited nearly an idea zero-order release pattern for the first 100 h. Meanwhile, the total release rate was further increased beyond 80% in the acidic solution containing GSH. However, the GDC NP control group showed insignificant difference among the four different conditions (**Figure 5B**). The total release amount of DOX was lower than 20% because the DOX was connected with PGal through covalent bonds that is, ester and amide bonds, and it showed insensitivity to GSH and low pH. Nevertheless, the DOX in the GDC NP group showed not only insensitive release feature but also fast release in all four conditions (**Figure 5C**). Considering that the DOX was non-covalently loaded at the hydrophobic core region of GDC NPs, the release of DOX was dependent on the free diffusion of DOX molecules from the inner core to the outer region, thereby leading to undesired release at pH 7.4. Moreover, the release rate of DOX was higher in acidic conditions than that at pH 7.4. This result is due to the fact that the hydrophobic DOX loaded at the core region becomes hydrophilic in low pH, thereby improving the release rate. These results demonstrated that GPD3 NPs possessed favorable responsive drug release characteristics, which was highly relative to the presence of GSH because the core region is cross-linked by disulfide bond. Even in an acidic medium, the release of DOX was limited by the shield of disulfide bond cross-linked network. Given that the endosomal environment is acidic, and the cytoplasm of cancer cells has a higher level of GSH (approximately 20-fold than that in normal cells), thereby exhibiting a reductive environment. These results indicated that GPD3 NPs may exhibit a specifically programmed release during the transportation in cancer cells.

### *In Vitro* Cytotoxicity Assay

**FIGURE 6:**
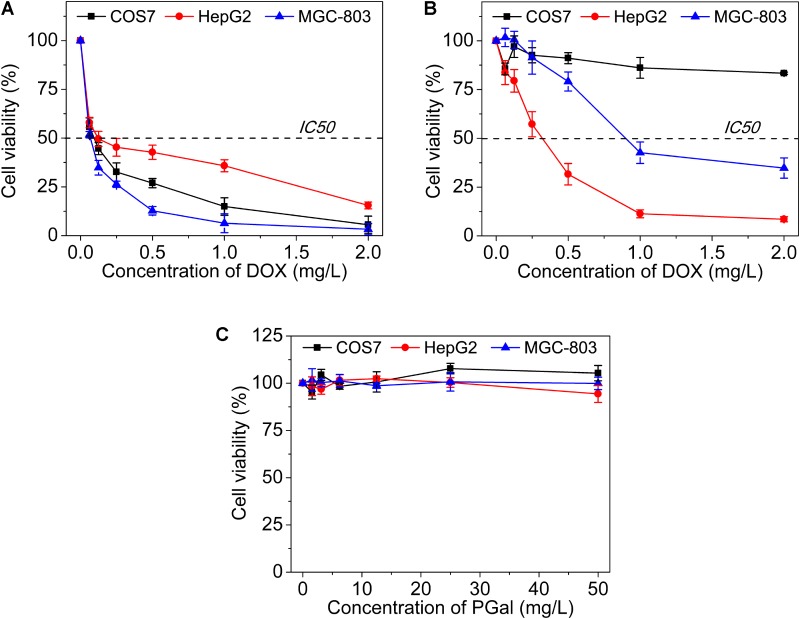
Cell viability of **(A)** free DOX, **(B)** GPD3 NPs, and **(C)** PGal against COS7, HepG2, and MGC-803 cells.

To evaluate the cytotoxicity of GPD3 NPs, we employed COS7, HepG2, and MGC-803 cells in the investigation, as shown in **Figure 6**. The IC50 values of free DOX against three types of cells were all lower than 0.25 mg/l (**Figure 6A**). We found that free DOX exhibited insignificant difference against three types of cells. In addition, the viability of HepG2 cells was higher than that of COS7 and MGC-803 cells. The chemotherapeutic agent can barely distinguish between the target and non-target cells. This result is the main reason why chemotherapy is always accompanied with serious systemic toxicity. However, our designed GPD3 NPs showed selective toxicity against the three aforementioned cells (**Figure 6B**). The IC50 value was higher than 2 mg/l against COS7 cells, but only 0.32 mg/l against HepG2 cells, and 0.89 mg/l against MGC-803 cells. The cell viability of COS7 cells were higher than those of HepG2 and MGC-803 cancer cells. In addition, HepG2 cells exhibited the highest sensitivity to GPD3 NPs among the cells employed. Considering that PGal was non-toxic (**Figure 6C**), the cell inhibition was highly derived from the conjugated DOX. These phenomena occurred because HepG2 cell is a typical type of HCC, on which a higher amount of ASPGR is exposed. However, MGC-803 cell is a kind of gastric carcinoma cell and COS7 cell is a fibroblast-like cell. These mean ASPGR is non-expressed on both MGC-803 and COS7 cells, thereby resulting in non-recognition of the designed GPD NPs. Therefore, GPD3 NPs can efficiently and selectively deliver anticancer drug DOX to HCC cells and protect normal and non-target cells, showing potential in HCC therapy.

### Cellular Uptake

**FIGURE 7:**
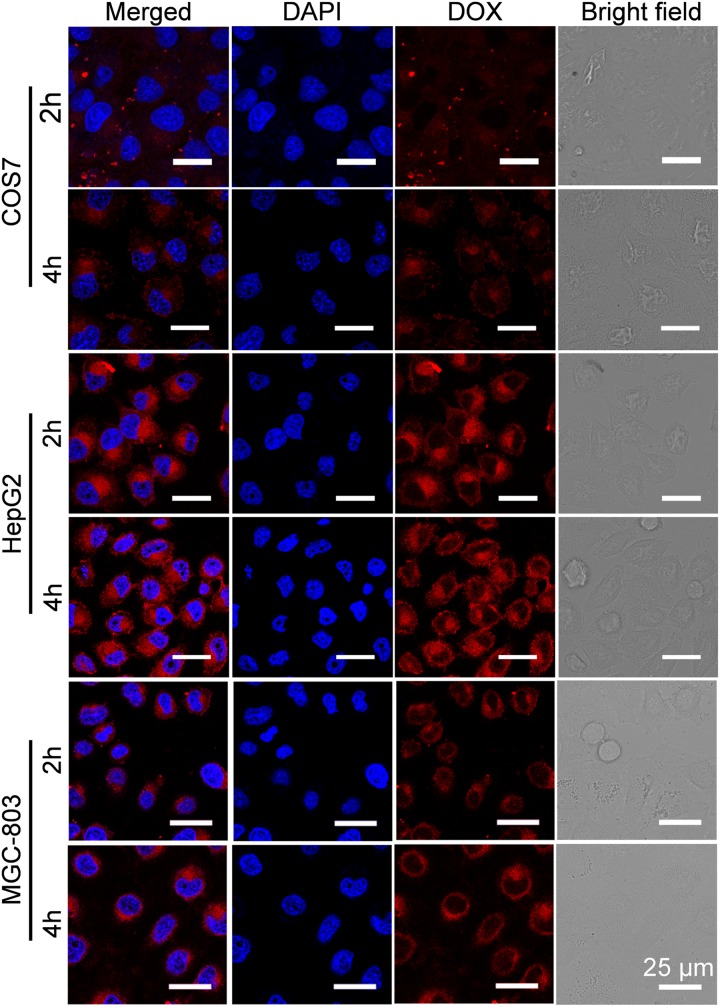
Cellular uptake of GPD3 NPs in COS7, HepG2, and MGC-803 cells after incubation of 2 and 4 h observed by CLSM. The cell nuclei were stained with blue probe DAPI.

**FIGURE 8:**
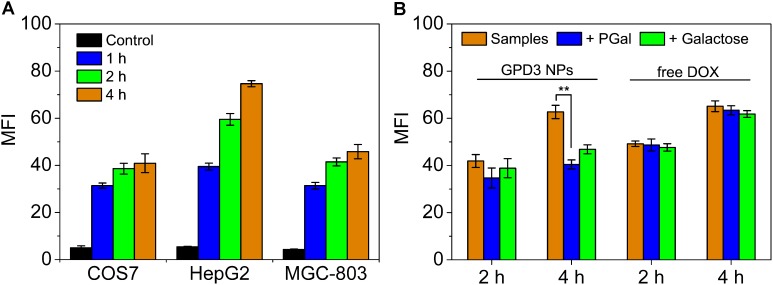
Mean fluorescent intensity (MFI) of **(A)** COS7, HepG2, and MGC-803 cells incubated with GPD3 NPs (equivalent to 10 mg/l of DOX) for different periods. **(B)** HepG2 cells incubated with GPD3 NPs (equivalent to 5 mg/l of DOX) and free DOX (5 mg/l) under the inhibition of galactose (2 mg/ml) and PGal (equibalent to 2 mg/ml of galactose). ^∗∗^*p* < 0.05.

To investigate the reason for the selective inhibition of GPD3 NPs against different cells, we used CLSM to observe the internalization of GPD3 NPs to different cells, as shown in **Figure 7**. Among these cells, we found brighter red fluorescence localized in HepG2 cells than those of COS7 and MGC-803 cells. Since red fluorescent DOX moieties were connected with PGal, these results showed that a higher amount of GPD3 NPs was internalized into HepG2 cells. Additionally, the red fluorescence in COS7 and MGC-803 cells showed insignificant variation with increasing incubation time. However, the fluorescent intensity was continuously increased in HepG2 cells. This phenomenon was due to the increasing amount of internalized GPD3 NPs and aggregation-caused quenching (ACQ) of DOX, which gathered at the hydrophobic core region of GPD3 NPs. As the DOX was gradually released into the cytoplasm of cells over the incubation time, the fluorescent intensity was increased with decreasing ACQ effect. These results were also consistent with the results in the drug release assay. We further used flow cytometry to quantitatively estimate the fluorescent intensity in different cells, as shown in **Figure 8A**. For the first 1 h, the MFI in three cells showed insignificant differences. However, the MFI value in HepG2 cells was increased with time and became nearly twofold when compared with that in COS7 and MGC-803 cells after 4 h. These results indicated that GPD3 NPs can selectively transport DOX to HepG2 cells and cause efficient cell inhibition. We hypothesized that this difference was due to the specific recognition between galactose, which coated on the surface of GPD NPs, and ASGPR exposed on HepG2 cells, thereby resulting in ASGPR-mediated internalization. Generally, ASGPR facilitates binding and uptake of circulating asialoglycoproteins through the recognition of the exposed terminal galactose (Lepenies et al., 2013). Nevertheless, autoimmune hepatitis, including HCC-induced inflammation, may lead to the suppression of ASGPR binding to asialoglycoproteins by the stimulatory effects of cytokines, such as interferon-γ, interleukin-2, and tumor necrosis factor, resulting in the exposure and increasing amount of ASGPR on the cell surface (Geijtenbeek and Gringhuis, 2009; Rigopoulou et al., 2012; Kuruvilla et al., 2017). Therefore, the designed GPD3 NPs, which were coated with galactose moieties on their surface, can be specifically internalized by HepG2 cells. However, the internalization of GPD3 NPs by COS7 cells and MGC-803 cancer cells was limited because ASGPR is not expressed on normal and gastric cancer cells, thereby showing a selective characteristic. To verify this hypothesis, we employed galactose and PGal as inhibition agents to decrease the internalization of GPD3 NPs in HepG2 cells (**Figure 8B**). We found that GPD3 NPs uptake was inhibited by both galactose and PGal. However, the internalization of free DOX was not influenced by these two inhibitors because they can bind with ASGPR and block the ASGPR-mediated pathway of GPD3 NPs. However, the inhibitor was unable to inhibit the free diffusion of the small molecular agent DOX. Given that the conjugated DOX was connected with GPD and encapsulated in the core of NPs, the above-mentioned results indicated that the drug was transported into the cells by the ASGPR-mediated pathway. We also found that PGal exhibited higher inhibition efficiency than that of galactose. This result was attributed to the documented cluster glycoside effect, which improved the binding ability of PGal to ASGPR (Dimick et al., 1999; Lundquist and Toone, 2002). These results not only indicated that our designed GPD NPs can be specifically internalized by HCC cells, but also revealed that glycopolymer-based GPD NPs can exhibit selective and enhanced internalization to HepG2 cells.

### Distribution Assay of GPD NPs

**FIGURE 9:**
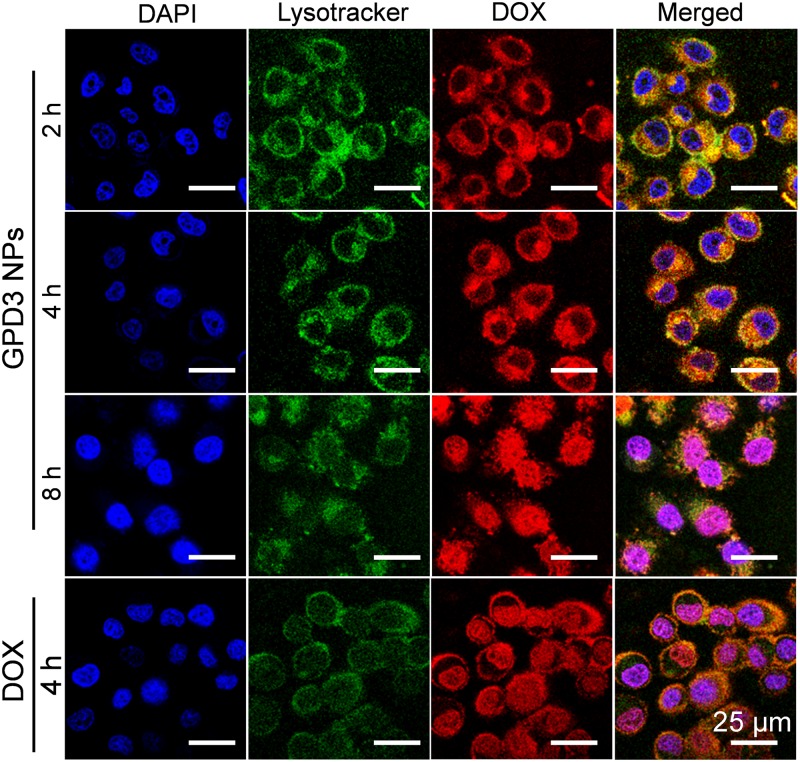
Cell distributions of GPD3 NPs (equivalent to 5 mg/l DOX) in HepG2 cells after incubation for different periods. Cell nuclei were stained with DAPI (blue), lysosomes, and endosomes were labeled with Lysotracker Green DND-26 (green). HepG2 cells incubated with free DOX (5 mg/l) was used as the control.

To observe the distribution of GPD3 NPs in HepG2 cells, we employed lysotracker as the fluorescent probe to label endosomes and lysosomes in the cells. As shown in **Figure 9**, we found that the intensity of red fluorescence was increased in cells for the first 2 and 4 h. However, the colocalization of red and green fluorescence indicated that GPD3 NPs were mainly distributed in endosomes and lysosomes for the first 2 h. These subcellular units are acidic; thus, NPs will enlarge and escape from the endosomes and lysosomes. Afterward, we found that the red fluorescence was distributed in cytoplasm after 4 h, which verified our hypothesis mentioned above. To the best of our knowledge, GSH is overexpressed in cytoplasm of cancer cells with both reducibility and acidity. Therefore, DOX would be rapidly released out from GPD3 NPs. Subsequently, red fluorescence was colocalized with blue fluorescence after 8 h, indicating that the drug was internalized into the cell nuclei stained with DAPI. These results demonstrated our designed GPD3 NPs exhibited a programmed drug transportation characteristic. For free DOX, the red fluorescence directly distributed in the cells and concentrated upon cell nuclei after coincubation for 4 h. This result was attributed to the fact that internalization of small molecular drug primarily depends on free diffusion, thereby showing rapid but non-selective characteristic.

## Conclusion

In summary, we have designed and prepared a series of dual-responsive GPD NPs for precise HCC therapy. GPD NPs possessed an adjustable size corresponding to the DL amount, thereby varying the hydrophilic/hydrophobic balance of amphiphiles. The model drug DOX was conjugated on the galactose-functionalized glycopolymer through the use of self-eliminating disulfide bond and boronate ester as linkages, thereby showing both redox-responsive and pH-sensitive characteristics. Moreover, the core cross-linking strategy stabilized GPD NPs in a normal physiological environment. However, performing rapid drug release feature in the milieu resulted in both reductivity and acidity. In addition, the transportation of the drug showed a programmed drug characteristic. Simultaneously, GPD NPs can be specifically internalized into HepG2 cells through an ASPGR-mediated pathway by the recognition of galactose, which coated on the surface of GPD NPs. Thus, these GPD NPs have potential uses in precise HCC therapy.

## Author Contributions

JW developed the main study. JY and BY helped in completing the synthesis. YW and ZX took part in the cell experiments and analysis. JhC and JxC drafted the manuscript and developed the study design. All authors have given final approval for this paper to be published.

## Conflict of Interest Statement

The authors declare that the research was conducted in the absence of any commercial or financial relationships that could be construed as a potential conflict of interest.
