# Supplementary material for: Dual-Responsive Core Crosslinking Glycopolymer-Drug Conjugates Nanoparticles for Precise Hepatocarcinoma Therapy
Source: Front Pharmacol. 2018 Jul 17;9:663. doi: 10.3389/fphar.2018.00663 (PMC6056621; doi:10.3389/fphar.2018.00663)
Supplement: Supplementary file 1 [file Presentation_1.pdf]

Supplementary Material

Dual-responsive core crosslinking glycopolymer-drug conjugates nanoparticles for precise hepatocarcinoma therapy

Jing Wu, Jiayi Yuan, Baotong Ye, Yaling Wu, Zheng Xu, Jinghua Chen*, Jingxiao Chen*

*** Correspondence:** Jinghua Chen: chenjinghua@jiangnan.edu.cn, Jingxiao Chen: tomchenjx@jiangnan.edu.cn.

1 Supplementary Data

1.1 Synthesis of PGal

The PGal was synthesized and identified by ^{1}H NMR.

GalAc-EMA (1). ^{1}H NMR (400 MHz, $CDCl_3$), δ (ppm): 6.13 (d, 1H), 5.59 (d, 1H), 5.20 (t, 1H), 5.08 (m, 1H), 5.01 (dd, 1H), 4.57 (d, 1H), 4.31 (d, 2H), 4.15 (s, 1H), 4.03 (d, 1H), 3.86 (d, 1H), 3.70 (d, 1H), 2.09 (s, 3H), 2.00 (s, 12H).

PGalAc (2) ^{1}H NMR (400 MHz, DMSO-*d6*), δ (ppm): 7.84 (s, 2H), 7.64 (s, 1H), 7.47 (s, 2H), 6.87 (s, 1H), 5.28 (s, 23H), 5.14 (s, 22H), 4.96 (s, 23H), 4.74 (s, 22H), 4.18 (s, 34H), 3.88 (s, 26H), 3.72 (s, 20H) 1.92-2.12 (m, 328H), 1.76 (s, 102H), 0.88 (d, 68H).

PGal (3) ^{1}H NMR (400 MHz, DMSO-*d6*), δ (ppm): 4.61 (d, 3H), 4.35 (s, 1H), 4.11 (d, 3H), 3.89 (s, 1H), 3.59 (d, 4H), 3.38 (d, 2H), 1.78 (s, 2H), 0.88 (d, 3H).

1.2 Synthesis of disulfide-activated DOX (DOX-ss-py)

1.2.1 Synthesis of 2-(pyridine-2-yl-disulfanyl) ethanol (4)

Briefly, 2-mercaptoethanol (0.42 mL, 5.74 mmol) was added dropwise into 15 mL of acetonitrile solution of methoxycarbonylsulfenyl chloride (0.55 mL, 6.04 mmol) under N_2 atmosphere. Afterwards, 6 mL acetonitrile solution of 2-mercaptopyridine (0.67 g, 6.03 mmol) was added with stirring at 0 ^{o}C for 30 min. The mixed solution was moved to room temperature with stirring for another 4 h. The precipitated product was filtered and washed with acetonitrile, and dried in vacuo. Yield: 94% (white solid). ^{1}H NMR (400 MHz, CD_3OD: $CDCl_3$=1:1): δ (ppm): 8.70 (m, 1H), 8.37 (m, 1H), 8.25 (m, 1H), 7.83 (m, 1H), 3.86 (m, 2H), 3.14 (m, 2H).

1.2.2 Synthesis of 2-[Benzotriazole-1-yl-(oxycarbonyloxy) ethyldisulfanyl] pyridine (5)

Triphosgene (0.49 g, 1.65 mmol) was dissolved in 20 mL of CH_2Cl_2 and degassed by N_2 for 10 min. Then, 2-(pyridine-2-yldisulfanyl) ethanol (1.12 g, 5.00 mmol) and 1.5 mL of triethylamine (TEA) was added under an ice bath and stirred at room temperature for 1.5 h. After that, 5 mL of CH_2Cl_2 solution containing 1-hydroxybenzotriazole (HOBt, 0.81 g, 5.99 mmol) and 0.75 mL TEA was added dropwise into the mixture. The reaction was stirred at room temperature for 16 h. Then, the reaction was quenched by adding DI water, and the solution was washed with 100 mL DI water and 100 mL NaCl

saturated solution, then, dried with anhydrous magnesium sulfate. The concentrated organic layer was grinded with hexane, and dried in vacuo to yield pure product. Yield: 63% (pale ashy solid). ^{1}H NMR (400 MHz, $CDCl_3$): δ (ppm): 8.48 (m, 1H), 8.22 (m, 1H), 8.02 (m, 1H), 7.78 (m, 1H), 7.73 (m, 2H), 7.56 (m, 1H), 7.12 (m, 1H), 4.82 (m, 2H), 3.28 (m, 2H).

1.2.3 Synthesis of disulfide-activated DOX (DOX-ss-py) (6)

DOX HCl (57.9 mg, 0.10 mmol) and 2-[Benzotriazole-1-yl-(oxycarbonyloxy) ethyldisulfanyl] pyridine (34.8 mg, 0.10 mmol) were dissolved in 15 mL of CH_2Cl_2. Then, 100µL DIEA (0.54 µmol) was added to the mixture. The mixture was stirred under N_2 atmosphere at room temperature for 18 h. After quenching the reaction by DI water, the solution was washed with DI water and saturated NaCl solution, then, dried with anhydrous magnesium sulfate. The solution was concentrated and poured into cold diethyl ether to obtain the precipitate. The red precipitation was collected by centrifugation, dried in vacuum. Yield: 94% (red solid, 6). ^{1}H NMR (400 MHz, $CDCl_3$): δ (ppm): 8.41(m, 1H), 7.77 (m, 1H), 7.64 (m, 1H), 6.82 (m, 1H), 4.75 (m, 2H), 3.65 (m, 2H).

1.3 Synthesis of glycopolymer-DOX conjugates (GPDs, 7)

GPD (7) ^{1}H NMR (400 MHz, DMSO-*d6*), δ (ppm): 7.83 (s, 5H), 7.52 (m, 4H), 7.31 (m, 2H), 6.74 (s, 2H), 6.66 (s, 1H), 4.76 (d, 17H), 4.57 (s, 8H), 4.39 (s, 7H), 4.15-3.99 (m, 36H), 3.62 (d, 33H), 2.87 (m, 4H), 1.92 (m, 12H), 0.86 (d, 23H).

2 Supplementary Figures

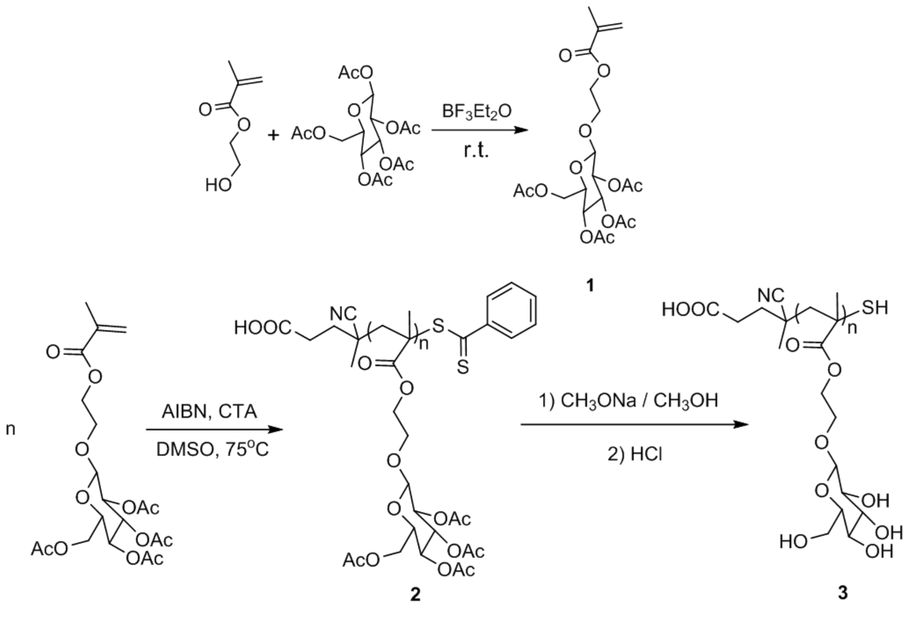

Supplementary Figure 1. Synthesis route of PGal.

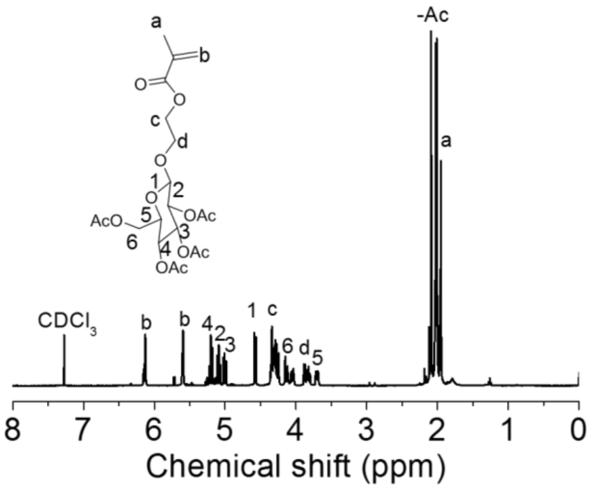

Supplementary Figure 2. ^{1}H NMR spectrum of GalAc-EMA (1).

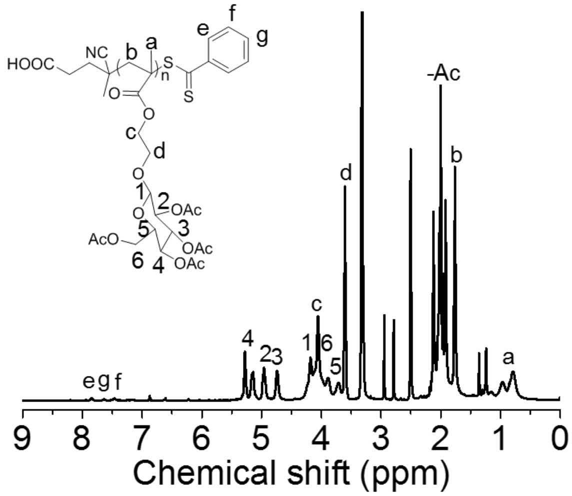

Supplementary Figure 3. ^{1}H NMR spectrum of PGalAc (2).

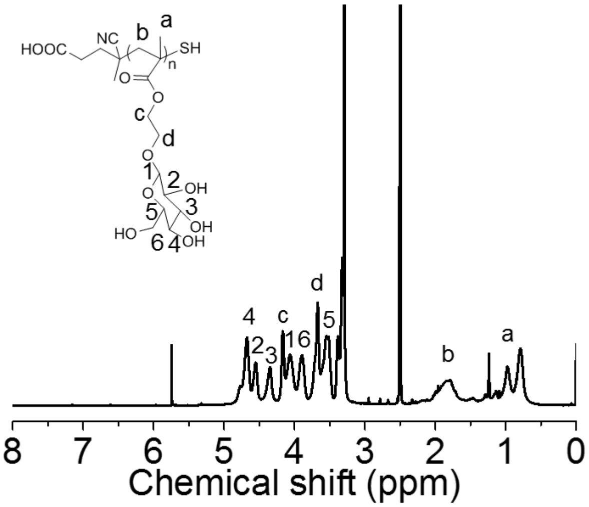

Supplementary Figure 4. ^{1}H NMR spectrum of PGal (3).

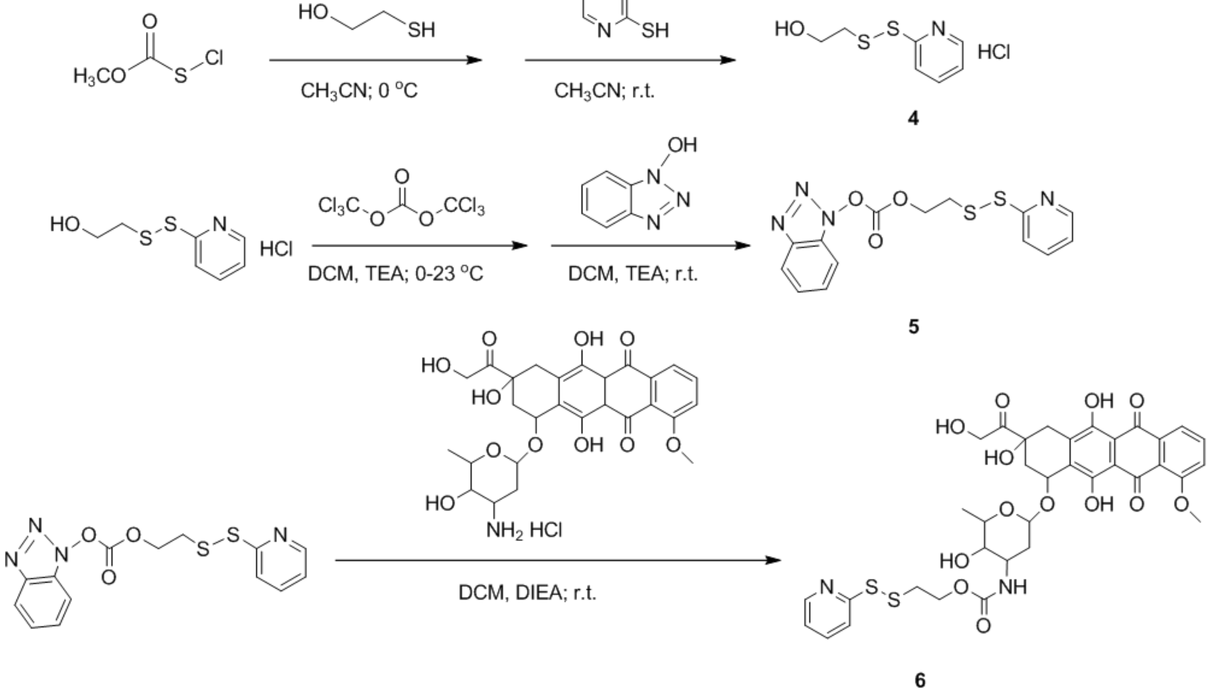

Supplementary Figure 5. Synthesis route of 2-(pyridine-2-yl-disulfanyl) ethanol (4) 2-[benzotriazole-1-yl-(oxycarbonyloxy) ethyldisulfanyl] pyridine (5) and DOX-ss-py (6).

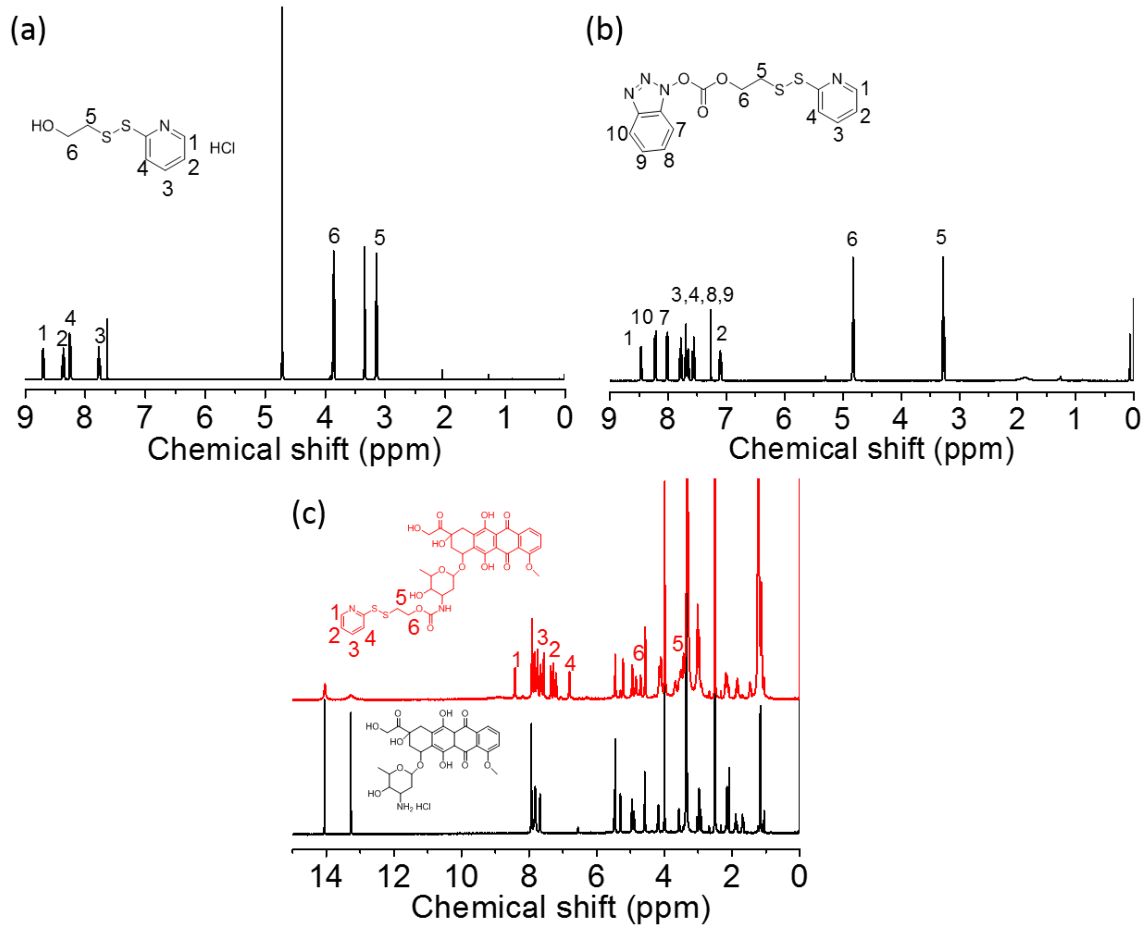

Supplementary Figure 6. ^{1}H NMR spectra of (a) 2-(pyridine-2-yl-disulfanyl) ethanol (b) 2-[Benzotriazole-1-yl-(oxycarbonyloxy) ethyldisulfanyl] pyridine and (c) DOX-ss-py.

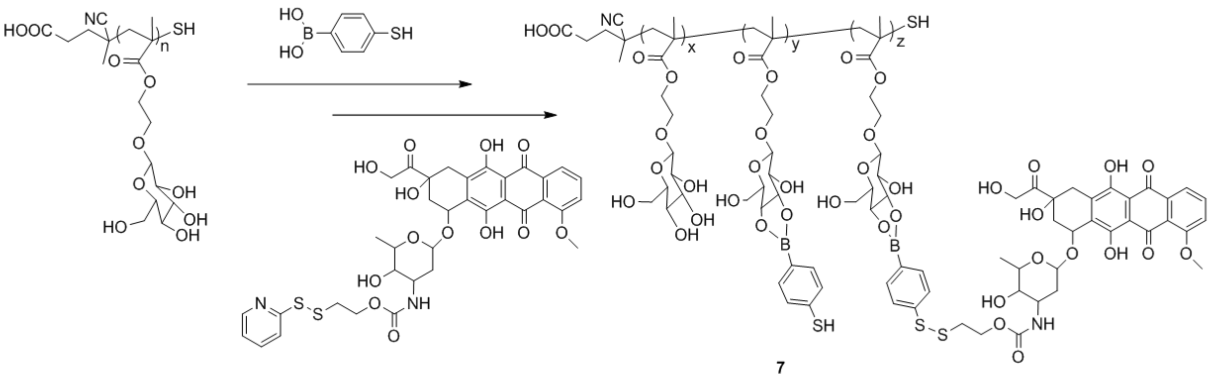

7

Supplementary Figure 7. Synthesis route of GPDs.

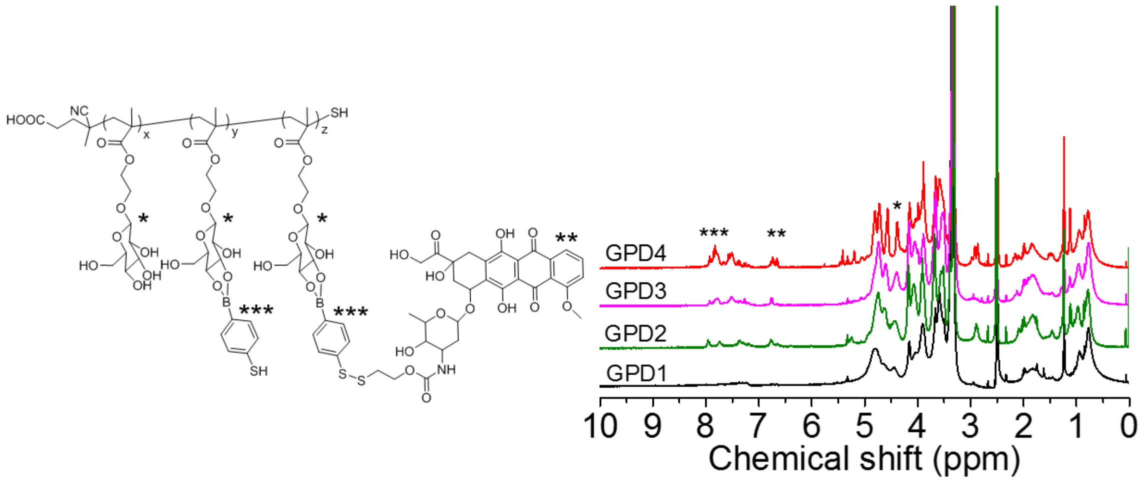

Supplementary Figure 8. ^{1}H NMR spectra of four GPDs.

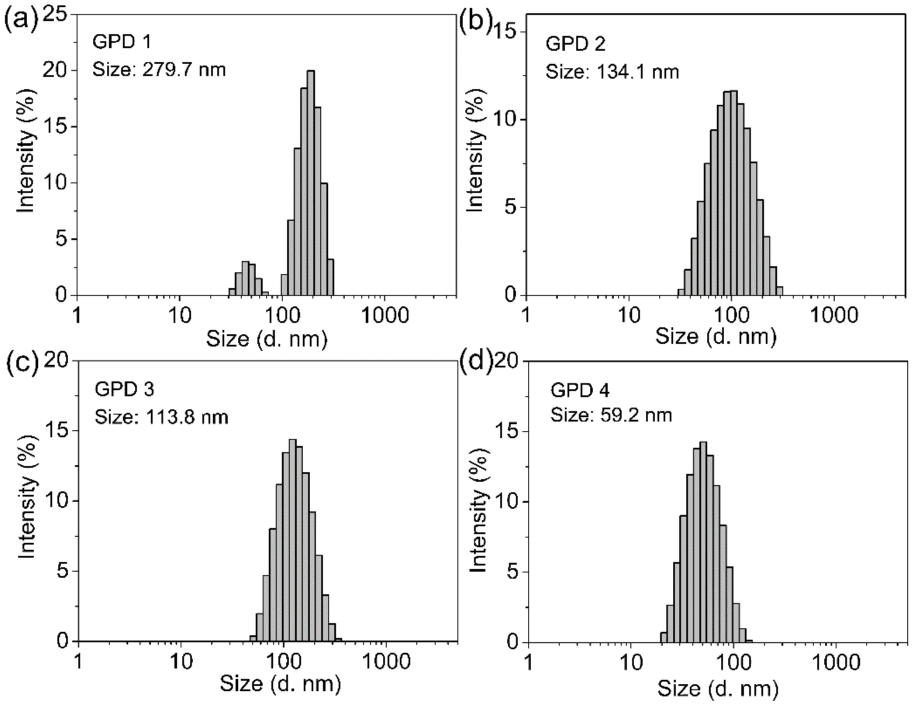

Supplementary Figure 9. Size distribution of GPD1, GPD2, GPD3 and GPD4 NPs in PBS (pH 7.4).

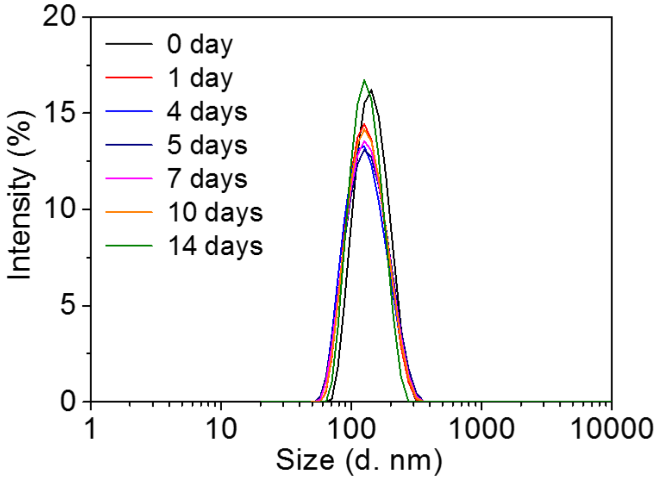

Supplementary Figure 10. Size distributions of GPD3 NPs in PBS (pH 7.4) during two weeks.
